# Investigation of Electrocatalysts Produced by a Novel Thermal Spray Deposition Method

**DOI:** 10.3390/ma13122746

**Published:** 2020-06-17

**Authors:** Walid Hetaba, Alexander Yu. Klyushin, Lorenz J. Falling, Dongyoon Shin, Anna K. Mechler, Marc-Georg Willinger, Robert Schlögl

**Affiliations:** 1 Department of Inorganic Chemistry, Fritz Haber Institute of the Max Planck Society, Faradayweg 4-6, 14195 Berlin, Germany; klyushin@fhi-berlin.mpg.de (A.Y.K.); frevel@fhi-berlin.mpg.de (L.J.F.); marc.willinger@scopem.ethz.ch (M.-G.W.); acsek@fhi-berlin.mpg.de (R.S.); 2 Department of Heterogeneous Reactions, Max Planck Institute for Chemical Energy Conversion, Stiftstr. 34-36, 45470 Mülheim an der Ruhr, Germany; shingoon86@gmail.com (D.S.); anna.mechler@cec.mpg.de (A.K.M.); 3 Division of Energy Materials, Helmholtz-Zentrum Berlin für Materialien und Energie GmbH, Albert-Einstein-Str. 15, 12489 Berlin, Germany

**Keywords:** supported catalysts, electrocatalysts, thermal spray deposition, electron microscopy, EDX, XPS, cyclo-voltammetry

## Abstract

Common methods to produce supported catalysts include impregnation, precipitation, and thermal spray techniques. Supported electrocatalysts produced by a novel method for thermal spray deposition were investigated with respect to their structural properties, elemental composition, and electrochemical performance. This was done using electron microscopy, X-ray photoelectron spectroscopy, and cyclic voltammetry. Various shapes and sizes of catalyst particles were found. The materials exhibit different activity towards oxidation and reduction of Fe. The results show that this preparation method enables the selection of particle coverage as well as size and shape of the catalyst material. Due to the great variability of support and catalyst materials accessible with this technique, this approach is a useful extension to other preparation methods for electrocatalysts.

## 1. Introduction

The activity of solid catalysts usually depends on the active surface area per unit volume of the catalytic material. Therefore, small catalyst particles are preferable [1]. At relevant working conditions, small particles of the active species alone are usually not stable enough. Thus, a supporting material is used to provide the stability, shape, and porous structure necessary, while the active phase provides the desired functionality [2]. In some cases, the support enhances the catalytic activity of the catalyst [3,4,5,6]. In order to gain high activity, a dense distribution of small particles of the active component over the surface of the support is desired [2,7]. However, the contact between the catalyst particles should be as limited as possible [1]. Additionally, it is important for electrocatalytic applications that the catalytic material is in good electrical contact with the support [4,8,9].

Reviews of different synthesis methods for electrocatalysts can be found in the literature, e.g., by Shao et al. [10] and Li et al. [11]. The performance of catalysts prepared by the reported methods is evaluated by their behavior in the oxygen reduction reaction. The synthesis procedures include microemulsion [12], (template based) impregnation [12,13,14], impregnation followed by lyophilization [15,16], precipitation [17], electrodeposition [18,19], chemical vapour deposition [20], a molecular encapsulation approach [21], magnetron sputtering [22], plasma assisted preparation methods [20,23], and ink-jet printing of a “catalyst ink” [24,25,26]. The most common methods to produce supported catalysts are impregnation and precipitation [1,27]. However, the preparation conditions of the nanoparticles strongly influence the morphology and composition of the particles, which in turn affects the electrocatalytic behavior of the material [28,29,30,31]. In this work, we focus on a thermal spray deposition method which belongs to a class of techniques that have advantages compared to the previously mentioned techniques, such as the low operation cost and the wide variety of metals that may be used. An overview of different spraying techniques was performed by Herman et al., including combustion flame spraying, high-velocity oxy-fuel (HVOF) spraying, two-wire electric arc spraying, plasma spraying, and cold-spraying techniques [32]. A thorough analysis of warm spraying techniques including the evaluation of different prepared samples in comparison to HVOF spraying is given by Kuroda et al. [33]. Moridi et al. published a review of cold spraying techniques and a detailed investigation of samples prepared by this coating method [34].

All the described methods have certain advantages and disadvantages and are suitable for specific tasks. A common challenge of all of these methods is upscaling and the use of different materials without large changes to the setup or synthesis route. Thus, we investigate a novel method to deposit catalyst nanoparticles on a metal support which is described in the patent [35] filed by TreadStone Technologies, Inc. (Princeton, NJ, USA). This technique overcomes some of the drawbacks of the previously mentioned deposition methods and has several advantages. The scope of the method reported in this provisional patent is the design of a cost-efficient technique to deposit nano- and micro-scale precious metal catalysts on a metal substrate as a support. It should be possible to design the catalyst material in such a way that it exhibits the desired catalytic activity and durability necessary for the applications in, e.g., fuel cells, using this method. The support material can have any desired size and shape (e.g., foil, plate, rod). Furthermore, a wide variety of (precious) metals can be used for deposition and the size of the resulting particles can be varied in a range of several nm to several μm. Additionally, the surface coverage by the catalyst particles can be varied using the described method.

In this work, we investigate four samples prepared by the thermal spray method described. The structural properties and elemental composition are analyzed and a first assessment of the electrochemical performance is given using electron microscopy, X-ray photoelectron spectroscopy and cyclic voltammetry.

## 2. Materials and Methods

### 2.1. Sample Preparation

We investigated four samples produced at TreadStone Technologies, Inc., each generated by different treatment according to United States patent US 9,765,421 B2 [35]. The samples are referred to using a unique number and, alternatively, a description according to the substrate and catalyst material used in the production process (Table 1). For sample “Au”, the catalyst material was deposited on one side of the support material, while for all other samples the deposition process was performed on both sides as a way to highlight the capability of the method to produce metal plates that are covered either on both sides with catalyst particles or on one side only.

The produced metal sheets were of 10 × 5 cm size. For X-ray photoelectron spectroscopy (XPS) and scanning electron microscopy (SEM) investigations, small pieces of approximatley 8 × 8 mm size were cut from the sheets. Using SEM, the area of interest for transmission electron microscopy (TEM) investigations was determined. Lamellae were cut from the investigated small pieces using Ga-ions in an FEI Helios focused ion beam (FIB) system to examine the material in TEM. Both sides of all specimen sheets were investigated using SEM and TEM.

### 2.2. Electron Microscopy

SEM was performed using a Hitachi S-4800 microscope (Hitachi High-Tech Europe GmbH, Krefeld, Germany), operated at an acceleration voltage in the range of 1–15 kV and a working distance between 2.5 and 10 mm. The images were recorded using a secondary electron (SE) detector. For energy dispersive X-ray (EDX) analysis, a Bruker silicon drift detector (SDD) (Bruker Corporation, Billerica, MA, USA) was used.

For TEM investigations, an FEI Talos F200X instrument (Thermo Fisher Scientific, Hillsboro, OR, USA) was used. This microscope is operated at 200 kV acceleration voltage and equipped with the SuperX EDX system, incorporating four SDDs. All investigations were performed using bright-field (BF) imaging as well as high resolution transmission electron microscopy (HRTEM). Additionally, scanning transmission electron microscopy (STEM) was used together with EDX analysis to investigate the elemental composition of the specimen. For all samples, EDX linescans and EDX maps of various regions were acquired. The FEI Ceta camera was used for BF and HRTEM imaging, while the Fischione high-angle annular dark field (HAADF) detector (E.A. Fischione Instruments, Inc., Export, PA, USA) with a camera length of 98 cm was used for STEM imaging.

### 2.3. X-ray Photoemission Spectroscopy

The XPS measurements were performed using a monochromatic Al Kα source (SPECS XR 50 (SPECS Surface Nano Analysis GmbH, Berlin, Germany)) and a hemispherical analyzer (SPECS Phoibos-Has 2500). All samples were mounted on a sapphire sample holder between a stainless steel back-plate and a lid with an 8 mm opening. All XP spectra were collected in normal photoemission mode. For quantitative XPS analysis, least-squares fitting of the spectra was performed using the CasaXPS software (version 2.3.18, Casa Software Ltd, Teignmouth, UK). A Doniach-Sunjic line shape was used to fit the Au 4f peaks, a Lorentzian asymmetric line shape was used to fit the Pt 4f and Ru 3d peaks and a Gaussian-Lorentzian product line shape was used to fit the C 1s, O 1s, Ti 2p, Zr 3d peaks. A Shirley-type background was subtracted before the spectra were fit.

### 2.4. Electrochemical Measurements

The ability to perform the oxidation of Fe (Fe${}^{+\mathrm{II}}$/Fe${}^{+\mathrm{III}}$) of each sample was measured using a three electrode cell connected to a potentiostat/galvanostat (BioLogic VMP3, BioLogic Science Instruments, Seyssinet-Pariset, France). A smooth Pt wire and Hg/HgO electrode were used as counter electrode and reference electrode, respectively. All potentials were measured versus Hg/HgO and then converted to reversible hydrogen electrode (RHE). Before measuring the activity of the sample in Fe oxidation, we applied a potential in the range of −0.4 to 0.1 V (vs. RHE) to remove surface impurities. The cyclic voltammograms were obtained at room temperature in Ar saturated 0.1 M KOH solution and also in a solution containing 1 mM $K_{4}\left[\mathrm{Fe}{\left(\mathrm{CN}\right)}_{6}\right]$ in 0.1 M KOH for the Fe redox measurements.

## 3. Results

### 3.1. Sample “Au”

**Figure 1:**
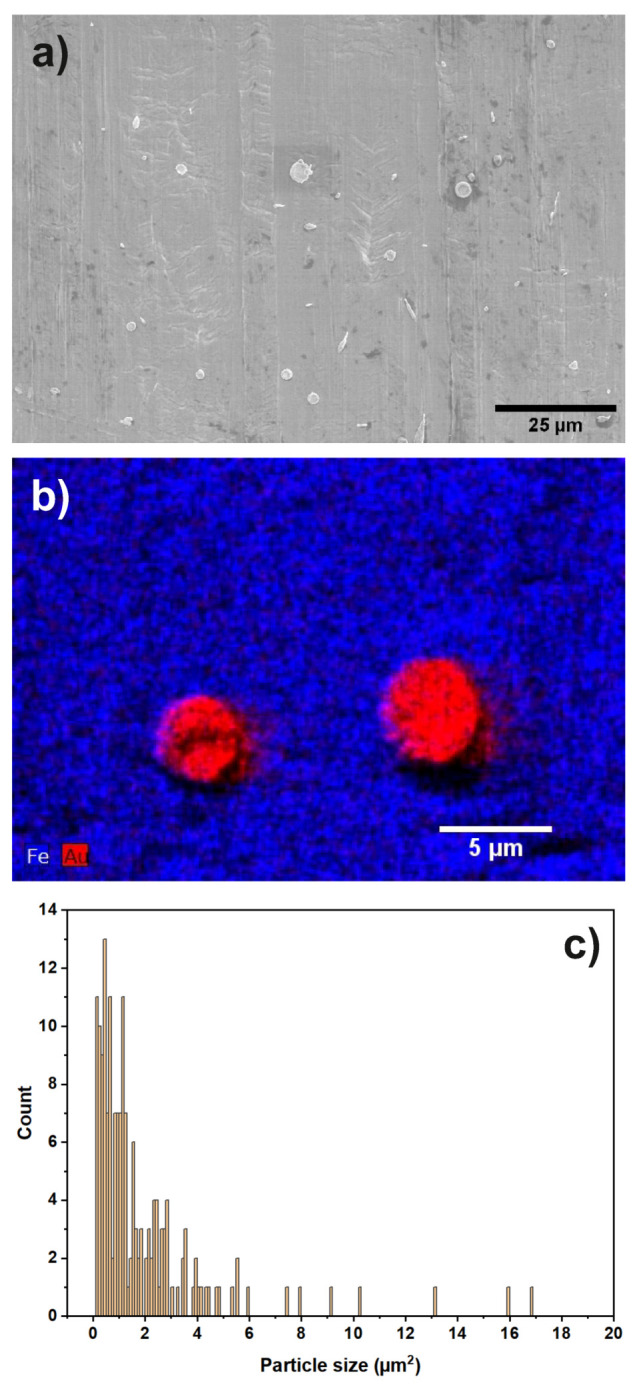
(**a**) SEM overview image of sample “Au”. Different shapes of Au particles (elongated, circular) are visible; (**b**) EDX map of two Au particles on the steel support; (**c**) particle size distribution of the catalyst particles.

In Figure 1a, an SEM overview image of sample “Au” is shown.

Au particles can be identified as bright regions on top of the steel substrate. The observed particles are of mixed morphology (circular, elongated and irregularly shaped). Figure 1b shows an EDX map focusing on two Au particles on the steel support. The size of the Au-particles is determined from the image as 3 to 5 μm. Inspecting several SEM images, an Au particle coverage, considering particles of at least 0.5 μm size, on the steel support of 0.57% is found. In Figure 1c, the particle size distribution is shown, determined from several SEM images. The additional SEM images used are given in the Supporting Information (Figure S1). An EDX map of a larger region can also be found in the Supporting Information (Figure S2).

**Figure 2:**
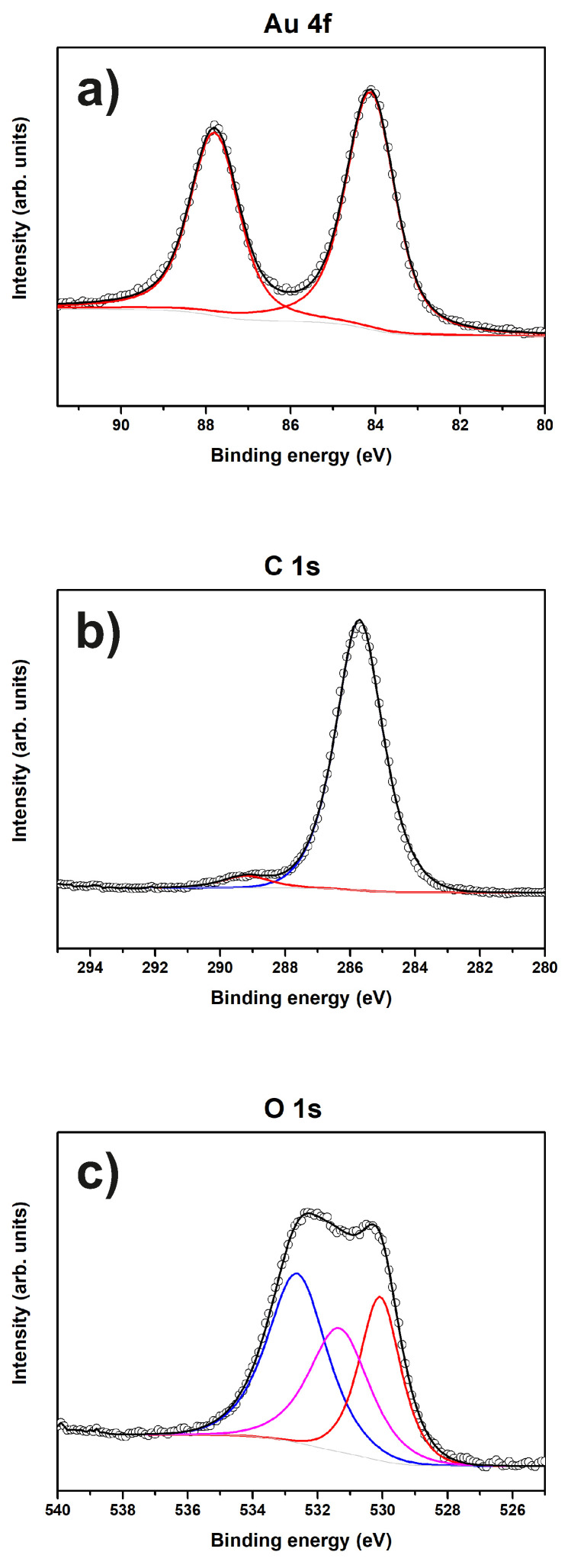
XP spectra of sample “Au”. The peaks of (**a**) Au 4f; (**b**) C 1s; and (**c**) O 1s and corresponding fits are shown.

Figure 2 shows the Au 4f, C 1s and O 1s XP spectra of sample “Au”.

The Au 4f spectrum shown in Figure 2a consists of only one component and can be assigned to metallic Au according to its binding energy (BE) of 84.1 eV [36]. The C 1s spectrum seen in Figure 2b is comprised of at least two components, the major peak at 285.7 eV associated with the contributions of both C-O and C-OH functionalities [37] and the following small peak at 289.3 eV which represents carboxyl groups [38]. The corresponding O 1s spectrum in Figure 2c consists of three components: the low BE peak at 530.1 eV is associated with lattice oxygen of ferric oxide (Fe${}_{2}$O${}_{3}$) from the steel substrate [39], while the peaks at 531.3 eV and 532.6 eV can be assigned to C=O and C-O, respectively [40,41,42].

**Figure 3:**
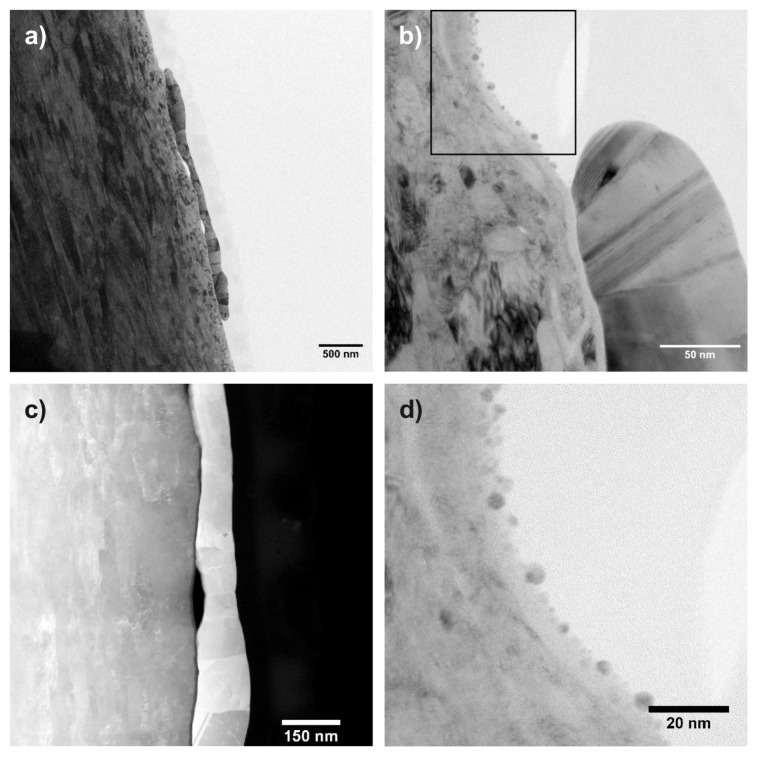
(**a**) bright field (BF) image of sample “Au”. A polycrystalline Au particle is visible in the middle part of the micrograph while on the left hand side the steel substrate can be seen; (**b**) details of the edge of the Au particle in higher magnification. The rectangle marks the area which was further digitally magnified; (**c**) STEM high-angle annular dark field (HAADF) image of the substrate and an Au particle; (**d**) digitally magnified part of the image marked in (**b**). Some Au nanoparticles are placed inside the oxide layer on top of the substrate.

Figure 3a shows a bright field (BF) TEM image of sample “Au”.

On the left-hand side, the stainless steel substrate is visible, while a polycrystalline Au particle can be seen in the middle part of the micrograph. A carbon protection layer used during the FIB preparation is discernible in very light contrast on top of the particle and the substrate. Figure 3b shows the edge of the Au particle in a higher magnification. An oxide layer between the substrate and the particle can be seen. Furthermore, the presence of Au nanoparticles in addition to the larger Au-particles was detected. In Figure 3c, a STEM high-angle annular dark field (HAADF) image of the substrate and a part of an Au particle is shown. During STEM acquisition, the images were rotated such that the interface is parallel to the vertical direction to facilitate comparison with the other samples. From the STEM image, it is evident that the substrate as well as the Au-particle are polycrystalline with differently oriented grains. The HAADF image gives Z-contrast, so it allows for distinguishing phases with different atomic numbers. Thus, it can be seen that a thin oxide layer and a cavity, which appear darker in the HAADF image, are located between the Au-particle (with higher Z) and the steel substrate (appearing slightly darker due to lower mean Z).

**Figure 4:**
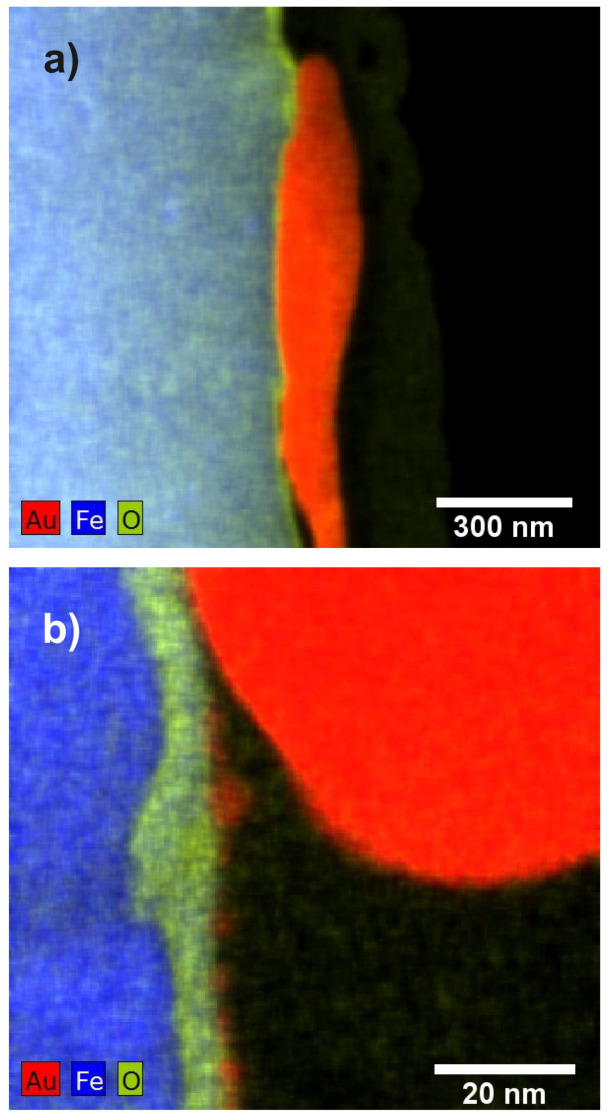
(**a**) STEM EDX mapping of the sample “Au”; (**b**) EDX mapping acquired with higher magnification showing an oxide layer on top of the steel substrate. Additionally to the edge of a large Au particle, Au nanoparticles are seen at the surface.

In Figure 4, the results of the STEM EDX mapping are shown.

**Figure 5:**
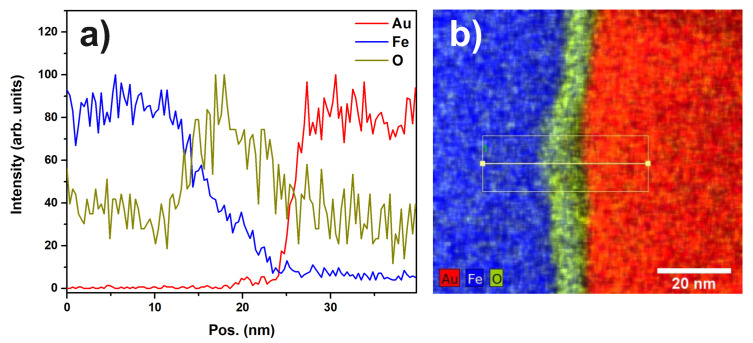
(**a**) EDX linescan of sample “Au” showing the intensities of the Au, Fe and O signals; (**b**) EDX mapping indicating the region from which the linescan was extracted.

A continuous oxide layer can be seen on top of the steel substrate. This layer is also present between the substrate and the Au particles. However, it is thinner in this region of the sample compared to the non-covered region of the substrate. Linescans such as the one shown in Figure 5 indicate that this oxide layer has a thickness of approximately 10 nm (FWHM of the oxygen signal). From the images, a thickness of up to 185 nm for the large Au particles and a size of 2–6 nm for the Au nanoparticles can be deduced.

**Figure 6:**
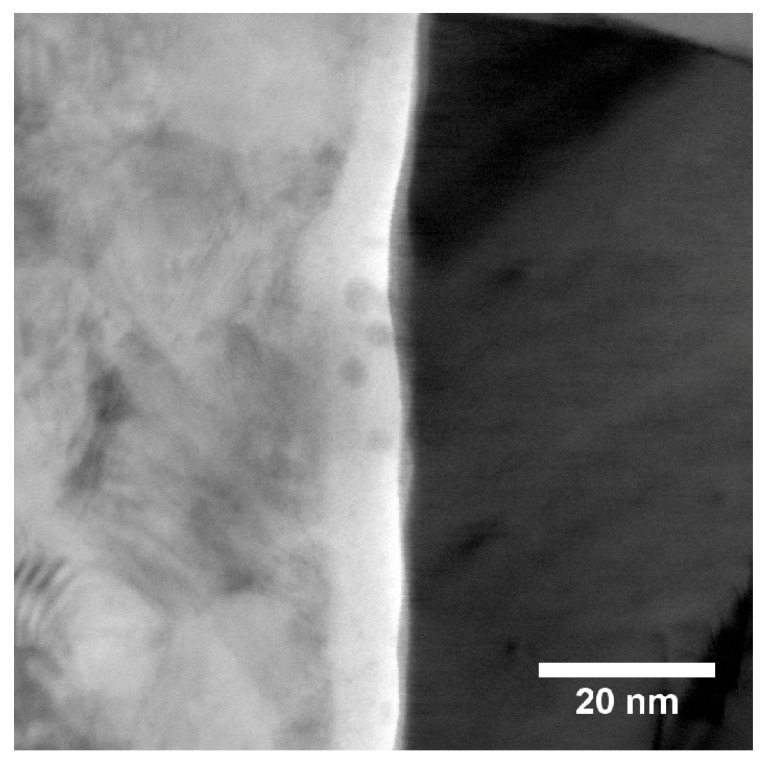
STEM BF image of sample “Au” indicating the presence of Au nanoparticles in the interfacial oxide layer between larger Au particles and the steel support.

In Figure 3d, it can be seen that some Au-nanoparticles are placed inside the oxide layer during the production process, thus establishing a conductive connection from the top of the sample to the steel substrate. Furthermore, the BF image in Figure 6 shows that some Au nanoparticles (size of ca. 5 nm) can be found in the interfacial oxide layer between the larger Au particles and the steel substrate. Additionally, smaller Au nanoparticles with a size of 1 to 2 nm were found in this region (see Supporting Information, Figure S3 for more details). This also indicates the possibility of electric conduction between the particles and the substrate.

### 3.2. Sample “Au/Pt”

**Figure 7:**
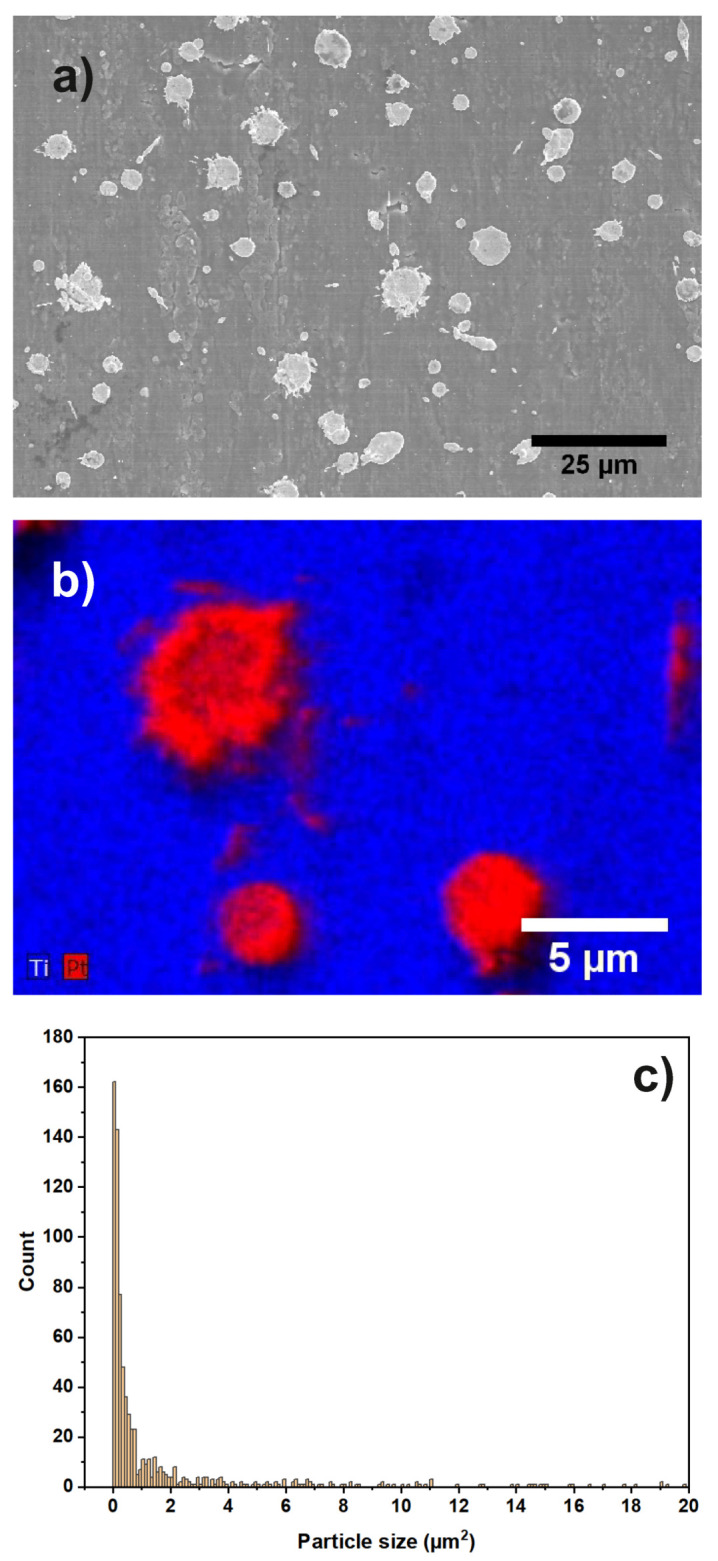
(**a**) SEM overview image of sample “Au/Pt”. Different shapes of Pt particles (elongated, circular, and irregularly shaped) are visible; (**b**) EDX map of several Pt particles on the Ti support; (**c**) particle size distribution of the catalyst particles.

The results of the SEM investigation of sample “Au/Pt” are shown in Figure 7. The particle shapes are again as expected when using a thermal spray deposition method.

The overview image shows Pt-particles of 0.5 to 5 μm size on top of the Ti substrate. Additionally, an EDX mapping of some particles is shown in higher magnification. From the mapping, it is clear that the particles consist of Pt, while the substrate contains Ti. The Pt particles cover 7.55% of the Ti support area according to SEM overview images. Figure 7c shows the particle size distribution, determined from several SEM images. The additional images are shown in the Supporting Information (Figure S4). An EDX map of a larger region is shown in the Supporting Information (Figure S5).

**Figure 8:**
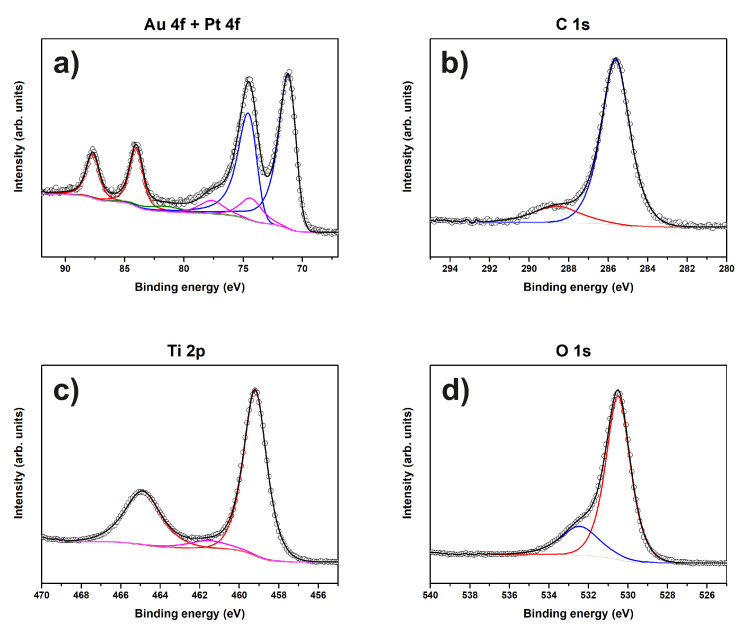
XP spectra of sample “Au/Pt”. The peaks of (**a**) Au 4f and Pt 4f, (**b**) C 1s, (**c**) Ti 2p and (**d**) O 1s and corresponding fits are shown.

In agreement with these measurements, the XPS data suggest that the sample consists of O and Ti as well as Pt and Au, and some C. Au4f, Pt4f, C1s, O1s and Ti2p XP spectra of sample “Au/Pt” are shown in Figure 8.

Similar to sample “Au”, only metallic Au is present on the surface (BE 84.0 eV). There are two Pt species at 70.8 eV and 74.3 eV, which are Pt${}^{0}$ and Pt${}^{4+}$, respectively [43,44]. The corresponding C1s spectrum shows two species, as in the spectrum of sample “Au”. The O1s spectrum is represented by C-O and TiO${}_{2}$ (BE 530.5 eV) [45,46] species. The presence of TiO${}_{2}$ on the surface is also confirmed by the Ti2p spectrum (Figure 8c), the BE of 459.2 eV matches the literature value [46].

**Figure 9:**
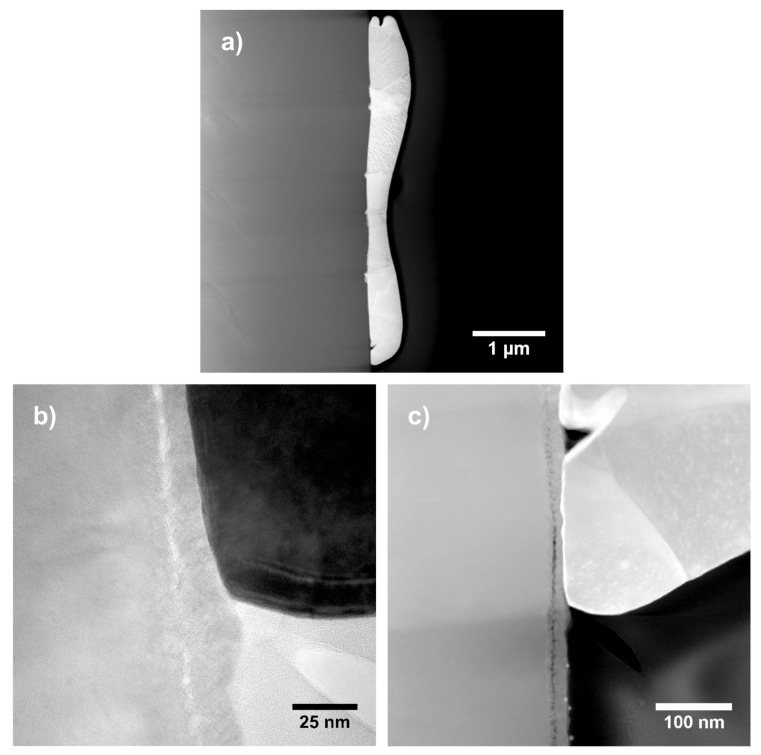
(**a**) STEM HAADF overview of Ti substrate and Pt particle in sample “Au/Pt”; (**b**) TEM BF image of the interface between Ti support and Pt catalyst particle. A 20 nm thick oxide layer is visible. The bright contrast between support and oxide layer indicates less densely packed grains or cavities; (**c**) STEM HAADF image of the edge of a Pt particle. The oxide layer is clearly visible between support and Pt particle. Regions of dark contrast at both interfaces of the oxide layer with the support and the Pt particle indicate regions of less electron scattering. At the bottom part, several 5 to 10 nm small Au nanoparticles can be seen on top of the oxide layer.

Figure 9a shows a STEM HAADF overview of the Ti substrate and a Pt particle.

**Figure 10:**
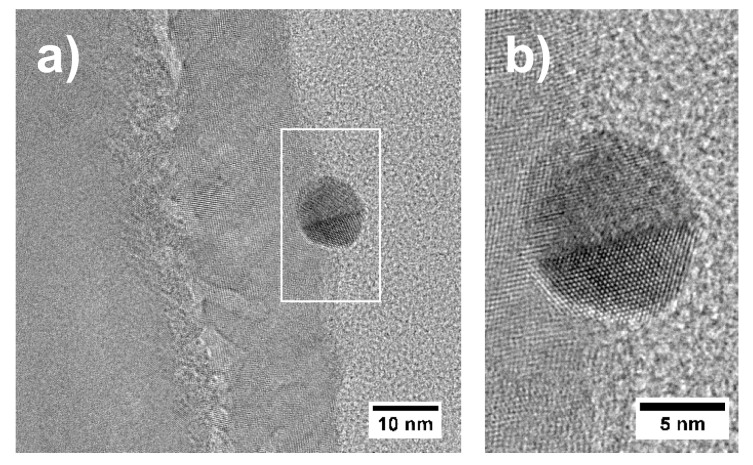
(**a**) HRTEM image of the oxide layer and an Au nanoparticle in sample “Au/Pt”. The frame indicates the region which was digitally magnified; (**b**) digitally magnified image of an Au nanoparticle indicating that these particles are polycrystalline.

The close-up TEM BF image in Figure 9b indicates the presence of a titanium-oxide layer with a thickness of up to 20 nm, which is in line with XPS results. The layer is found on top of the Ti substrate and between the substrate and the Pt particles. The images in Figure 9b,c suggest that there is a thin layer between the substrate and the oxide layer, which leads to less scattering of the electrons. This is indicated by the brighter contrast at the interface in the TEM BF image (Figure 9b) and the dark contrast at the interface in the STEM HAADF image (Figure 9c). Comparing with an EDX linescan (Supporting Information, Figure S6), this thin region shows a dip in the overal EDX intensity. The dark region between the Pt particle and the oxide layer in the upper part of Figure 9c, however, indicates a cavity. Furthermore, it is evident that the Pt particles are polycrystalline. Additionally, in Figure 9c, the presence of 5–10 nm large nanoparticles can be seen. In contrast to the larger Pt particles, these small nanoparticles are identified as Au particles using EDX. This is in agreement to the nominal composition of this sample which states Ti for the support and Au as well as Pt as catalyst material. Figure 10 shows a HRTEM image of one of these Au nanoparticles, giving evidence of the fact that these particles are also polycrystalline.

**Figure 11:**
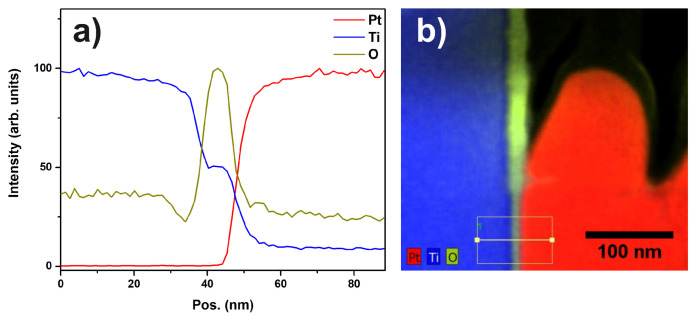
(**a**) EDX linescan of sample “Au/Pt” showing the signal intensities of the Pt, Ti and O peaks. The FWHM of the O signal indicates a thickness of 10 to 20 nm for the oxide layer; (**b**) STEM-EDX map showing the edge of a Pt particle, the oxide layer, and the Ti support. Additionally, the marker indicates the region from which the linescan was extracted.

The EDX map in Figure 11 and the corresponding linescan indicate that the layer of titanium-oxide has a thickness of about 10 to 20 nm.

**Figure 12:**
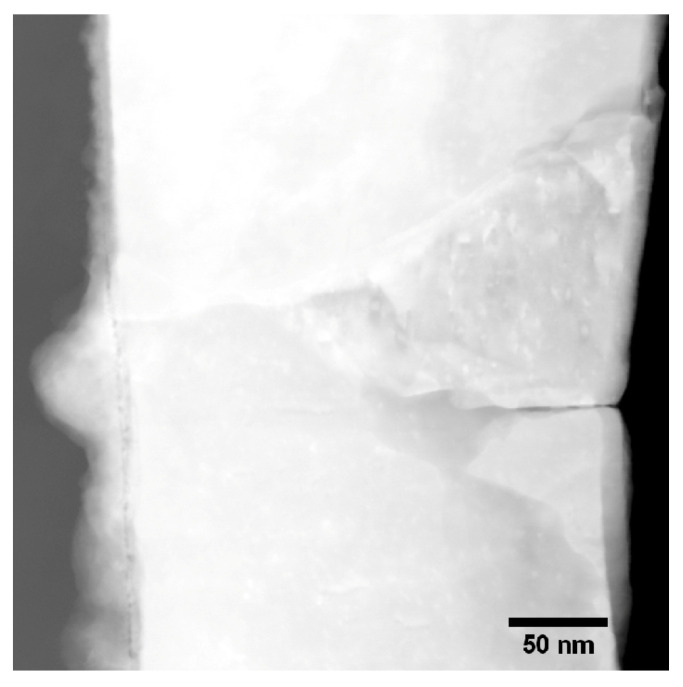
STEM HAADF image of a Pt particle and the Ti support in sample “Au/Pt”. In the left part of the image the diffusion zone in which Pt is diffusing into the substrate is visible.

**Figure 13:**
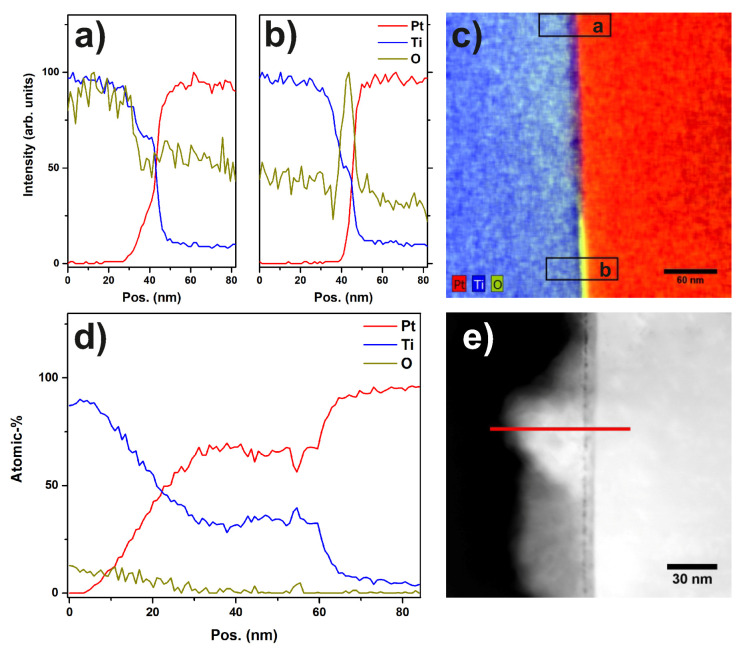
(**a**,**b**) EDX linescans across the interface between Pt catalyst particle and Ti substrate; (**c**) EDX map of the interface region with markers indicating the position of the extracted linescans; (**d**) EDX linescan of the diffusion zone at the interface between support and Pt catalyst particle. The atomic concentrations of Pt, Ti and O are shown; (**e**) STEM HAADF image indicating the region in which the linescan was recorded.

In Figure 12, a STEM dark field (DF) image of a Pt particle and the substrate is shown. At the very left part of the image, the diffusion of Pt into the substrate can be seen as cloudy structures. It is evident that the Pt diffuses across the interface layer, which is still visible in the diffusion zone. The interface layer can be identified by the cavities appearing darker in the DF image. Figure 13a,b show EDX linescans acquired across the interface between the Pt catalyst particle and the Ti substrate. The positions of the extracted linescans are marked in the EDX map in Figure 13c. The linescan and the EDX map show that the oxygen content of the interfacial layer is diminished in the region of diffusion compared to the non-diffusion zone. This indicates a conductive connection of the Pt catalyst particle and the Ti substrate in the diffusion zone. Taking a closer look at the diffusion zone, Figure 13d shows an EDX linescan of the marked region in Figure 13e. Again, the diminished O content in the interface layer is evident.

### 3.3. Sample “Ru/Ti-1”

**Figure 14:**
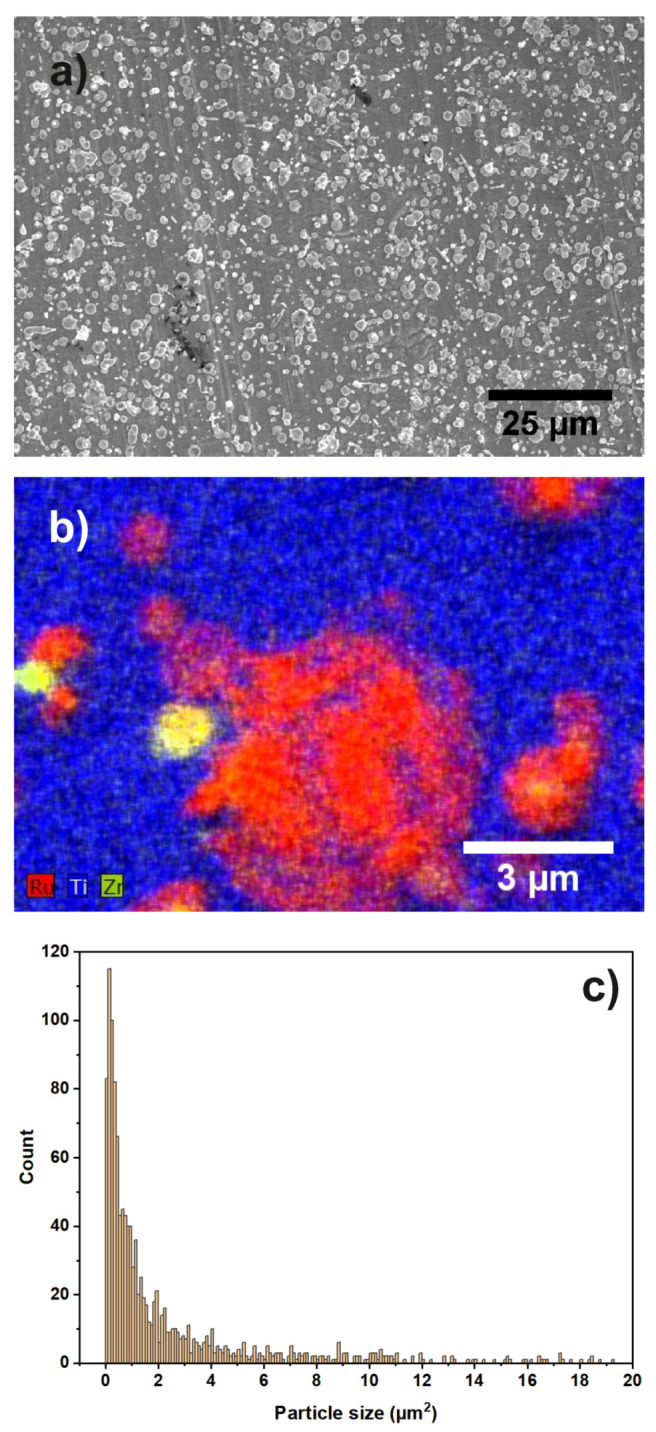
(**a**) SEM overview image of sample “Ru/Ti-1”. Different shapes and sizes of catalyst particles are visible; (**b**) EDX map of several Ru and Zr particles on the Ti support; (**c**) particle size distribution of the catalyst particles.

Figure 14a,b show an overview image and an EDX map of sample “Ru/Ti-1” acquired by SEM.

It can be seen that the substrate contains Ti, while the particles are composed of Ru and Zr. The nominal layer sequence of the produced sample states that the substrate is a Ti covered stainless steel sheet with Ru particles on top of it. The Zr is a residue from the ball milling process used during the preparation of the materials. From Figure 14b, it is evident that not all the Ru particles are covered by Zr. It was calculated from the overview images that the catalyst particles cover an area of 23.75% on the Ti support. In Figure 14c, the particle size distribution extracted from the SEM image in Figure 14a is shown. An EDX map of a larger sample region is shown in the Supporting Information (Figure S7).

**Figure 15:**
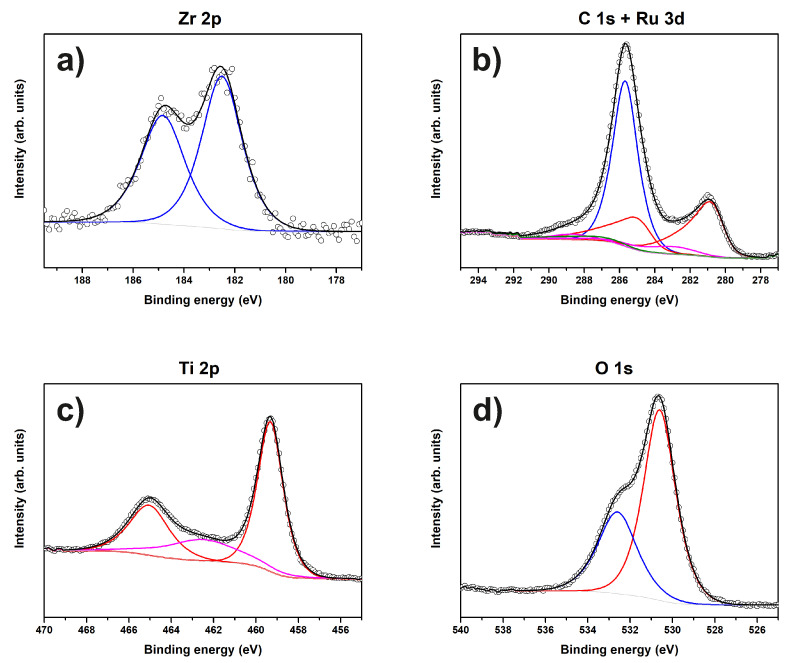
XP spectra of sample “Ru/Ti-1”. The peaks of (**a**) Zr 2p, (**b**) C 1s and Ru 3d, (**c**) Ti 2p, and (**d**) O 1s and corresponding fits are shown.

Figure 15 shows Zr 2p, C 1s, Ru 3d, O 1s, and Ti 2p XP spectra of sample “Ru/Ti-1”. The Zr 3d spectrum is fitted by a single component (ZrO${}_{2}$) with a BE of 182.5 eV [47]. The carbon species present in this sample are the same as for sample “Au/Pt”, namely C-O and carboxyl groups at 285.7 eV and 288.7 eV, respectively. RuO${}_{2}$ is present on the surface according to its BE of 280.6 eV [48,49]. The O1s spectrum consists of C-O and TiO${}_{2}$ components. No signal from the steel substrate is detected by XPS.

**Figure 16:**
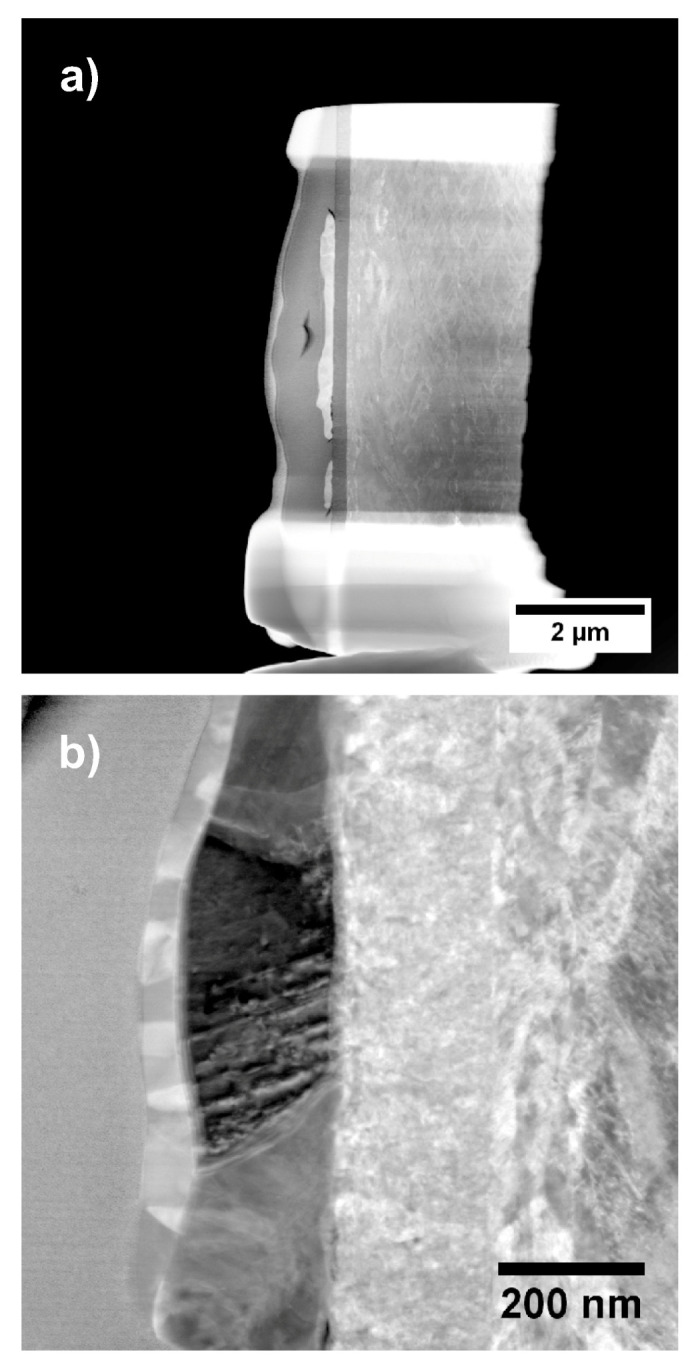
(**a**) STEM HAADF Overview image of sample “Au/Pt”. The layer structure consisting of different elements is easily seen due to the Z-contrast in the micrograph; (**b**) STEM DF detail image of a catalyst particle on the support. The different layers are (right to left): stainless steel, Ti, Ru, Zr, and Pt, which is used in the FIB preparation.

A STEM-HAADF overview image and a detail of the same region in higher magnification are shown in Figure 16a,b.

**Figure 17:**
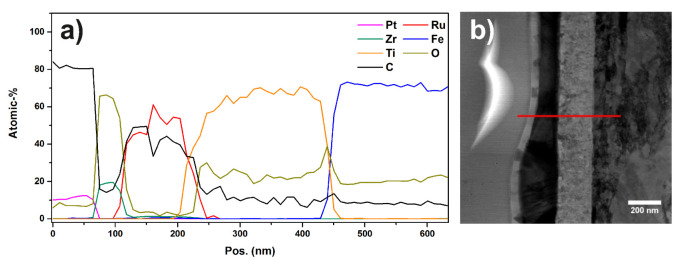
(**a**) EDX linescan of sample “Ru/Ti-1” showing the signal intensities of different elemental peaks. The layer structure is evident by inspecting the intensity ratios; (**b**) STEM DF image, the marker indicates the region in which the linescan was recorded.

The layer stacking of stainless steel, Ti, Ru, and Zr can be seen from the Z-contrast in the images and in the EDX-linescan of Figure 17 as well. Inspecting Figure 14b, it can be seen that not all particles are covered with Zr. Furthermore, if a particle is covered with Zr, the coverage is not necessarily stretched across the full area of the particle. The EDX-linescan shown in Figure 17 indicates the following stacking order of the layers and their thickness (from right to left): a substrate made of stainless steel is covered with a Ti layer which is approximately 220 nm thick, followed by the Ru particles which have a thickness of up to 200 nm.

Additionally, some of the Ru particles are covered with a Zr layer of about 30 to 50 nm thickness. Inspecting the EDX spectra of each layer (Supporting Information, Figures S8 and S9) in addition to the linescan, it is found that the Ti layer as well as the Zr layer contains a substantial amount of oxygen. A quantitative analysis using the PB/ZAF algorithm [50] reveals an elemental ratio of Ti/O as approximately 4/1. Furthermore, the topmost layer is identified as zirconia (ZrO${}_{2}$). Additionally, there is a very thin (approximately 5 nm) oxide layer between the steel substrate and the Ti layer.

### 3.4. Sample “Ru/Ti-3”

**Figure 18:**
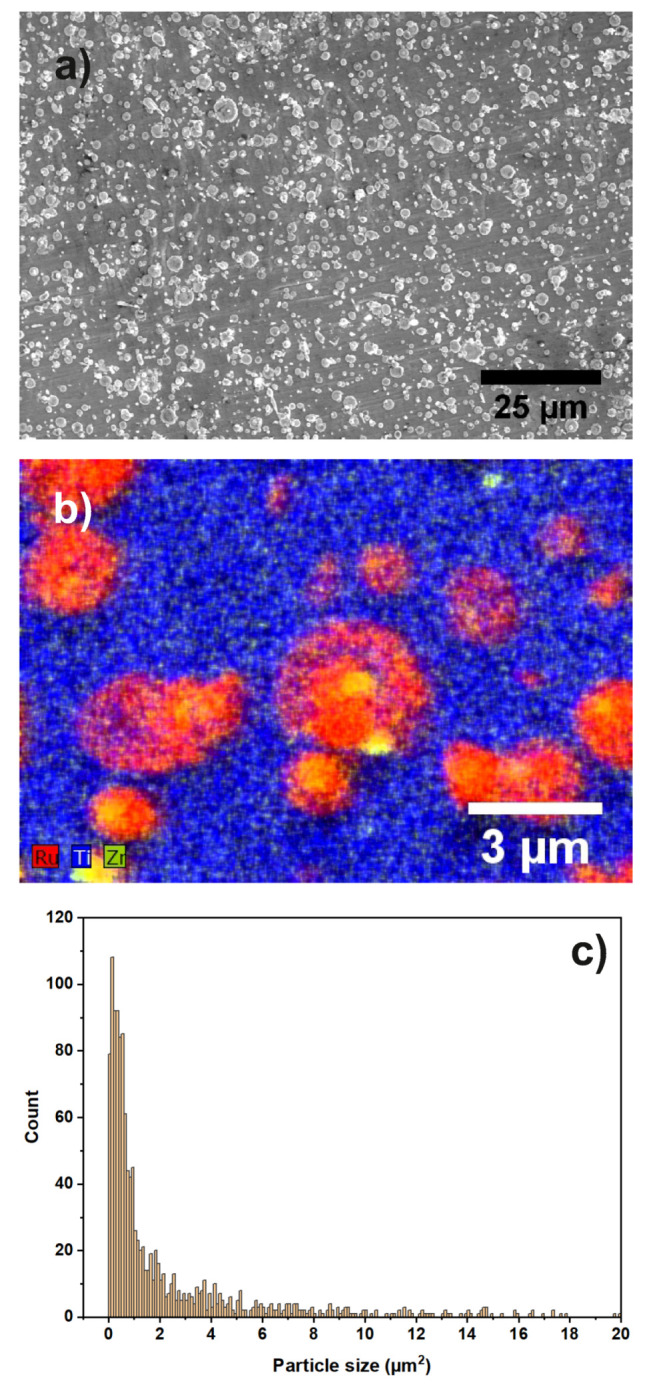
(**a**) SEM overview image of sample “Ru/Ti-3”. Different shapes and sizes of catalyst particles are visible; (**b**) EDX map of several Ru and Zr particles on the Ti support; (**c**) particle size distribution of the catalyst particles.

The layer structure of the sample “Ru/Ti-3” is the same as for the previous sample “Ru/Ti-1”. As can be seen from the SEM images and EDX-map shown in Figure 18a,b, the morphological structure is similar, with Ru particles, partly coverd with Zr, on top of a Ti covered steel substrate. The Zr is again a residue from the ball milling process during material preparation. An EDX map of a larger sample region can be found in the Supporting Information (Figure S10).

**Figure 19:**
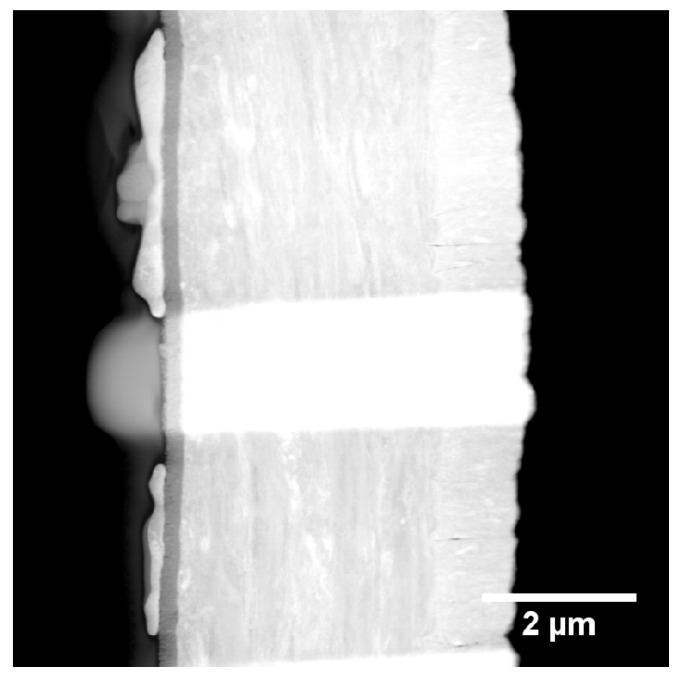
STEM HAADF overview image of sample “Ru/Ti-3”. The FIB preparation was performed in two regions with differently shaped catalyst particles.

**Figure 20:**
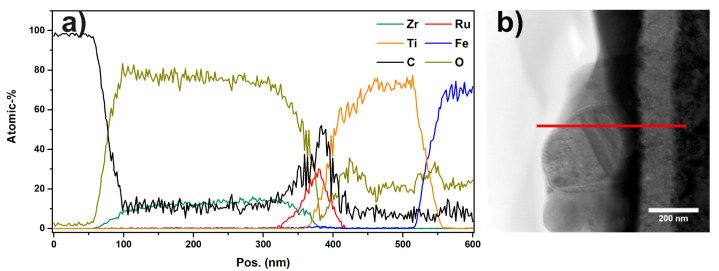
(**a**) EDX linescan of sample “Ru/Ti-3” showing the signal intensities of different elemental peaks. The layer structure is evident by inspecting the intensity ratios; (**b**) STEM DF image, the marker indicates the region in which the linescan was recorded.

Similarly to sample “Ru/Ti-1”, the catalyst particles cover an area of 23.17% of the support. In Figure 18c, the particle size distribution extracted from the SEM image in (**a**) is shown. The STEM HAADF overview image in Figure 19 shows the steel stubstrate and the Ti layer. Furthermore, it can be seen that the FIB cut was performed in two regions, one with a larger amount of Zr on top of the Ru particle and another one with a very low amount of Zr. The STEM image and the EDX-linescan of the Zr-rich region (Figure 20) indicate that the ZrO${}_{2}$ layer is up to 250 nm thick at its maximum, while the Ru layer has a thickness of only 50 nm in this region. The Ti layer is of 120 to 150 nm thickness. Other positions on this FIB lamella show that the Ru-particles can reach up to 200 nm thickness.

In all investigated areas of this sample, an oxide layer is found between the steel substrate and the Ti layer. In addition, a substantial amount of oxygen can be detected in the Ti and Zr layers. Quantitative analysis leads to the same elemental compositions as for sample “Ru/Ti-1”. Compared to the results of the previous sample, it is obvious that the interfaces in this sample are not as smooth and straight as in the previous sample.

The XPS measurement of sample “Ru/Ti-3” (Supporting Information, Figure S11) lead to the same results as for the previously described sample “Ru/Ti-1” and are in accordance to SEM and TEM results. For a more detailed description of the XPS measurements, the reader is referred to Section 3.3.

## 4. Discussion

Comparing all the experimental results for the four samples, we can find the following behavior of the supported catalysts: SEM and TEM images show that the production process yields nanoparticles of different size (up to 5 μm) and shape on top of a steel or Ti substrate (samples “Au” and “Au/Pt”, respectively) or on top of a titanium oxide layer (samples “Ru/Ti-1” and “Ru/Ti-3”). As expected, the morphology of the particles is therefore comparable to other materials generated by thermal spray deposition methods [32,33,35]. Furthermore, sample “Au/Pt” shows nm-sized Au nanoparticles on top of the Pt catalyst particles, as stated in the patent [35], and additionally on top of the substrate. In contrast to other reports about thermal spray deposition methods used to produce electrodes or solid oxide fuel cells [51,52], in samples “Au” and “Au/Pt”, an oxide layer is present at the interface between substrate and some of the catalyst particles (e.g., seen in Figure 4). Some catalyst particles exhibit diffusion of the catalyst material into the substrate (e.g., in Figure 12). Furthermore, some of the small nanoparticles are located inside the oxide layer between substrate and catalyst (Figure 6 and Figure S3). In the diffusion zone, the O content of the interfacial layer is reduced (see, e.g., Figure 13). The diffusion of catalyst material into the support and across the oxide layer facilitates conductive connection and thus electrochemical activity.In samples “Ru/Ti-1” and “Ru/Ti-3”, a very thin oxide interface layer can be found between the steel substrate and the Ti layer. The catalyst particles, however, are in direct contact with the Ti layer. Despite the similar composition of samples “Ru/Ti-1” and “Ru/Ti-3”, it can be seen that the surface of sample “Ru/Ti-3” exhibits a much higher roughness.

Comparing the results of the XPS measurements with the SEM and TEM measurements, we find that the elemental composition is coherent on the different scales investigated by the different methods. Furthermore, the XPS results allow the identification of different carbon containing groups, metal and oxide components. As the catalyst particles only partly cover the surface, signals from the substrate are also detected by XPS. However, as the probing depth does not exceed 20 nm, no signal of the steel substrate is detected for samples “Ru/Ti-1” and “Ru/Ti-3” as the Ti layer has a thickness of up to 220 nm and 150 nm, respectively.

**Figure 21:**
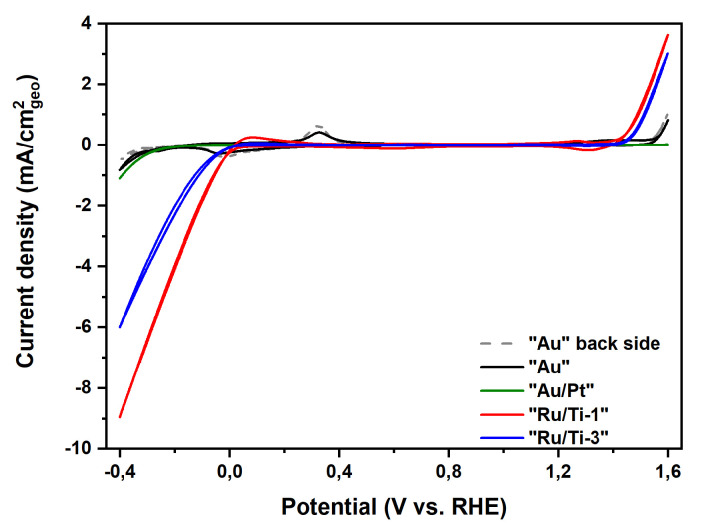
Cyclic voltammograms of all investigated samples obtained at a scan rate of 20 mV/s in Ar saturated 0.1 M KOH.

Cyclo-voltammetric measurements allow a first assessment of the catalytic activity of the four samples. Figure 21 shows the cyclic voltammograms of all four investigated samples in 0.1 M KOH electrolyte.

**Figure 22:**
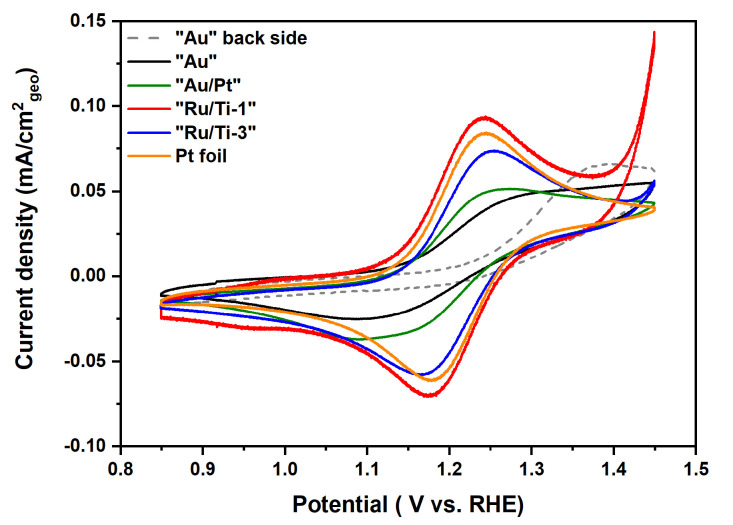
Cyclic voltammograms of all investigated samples obtained at a scan rate of 20 mV/s in a solution containing 1 mM $K_{4}\left[\mathrm{Fe}{\left(\mathrm{CN}\right)}_{6}\right]$ in 0.1 M KOH.

The catalytic activity for both the hydrogen evolution reaction (HER) and the oxygen evolution reaction (OER) can easily be confirmed. However, due to the low amount of active material, samples “Au” and “Au/Pt” show only little activity. It has to be noted that the back side of sample “Au” consists of pure stainless steel. The strong influence by the support is also reflected by two redox peaks in the region from −0.1V to 0.4V, which can be assigned to the oxidation/reduction of Fe${}^{+\mathrm{II}}$↔ Fe${}^{+\mathrm{III}}$ in stainless steel (see Supporting Information, Figure S12) [53]. Samples “Ru/Ti-1” and “Ru/Ti-3” show a much higher activity for both reactions compared to the other measured samples. Particularily, “Ru/Ti-1” shows a higher activity for HER and OER compared to “Ru/Ti-3” despite the similar coverage of the surface with Ru catalyst particles. This result leads to the assumption that the electrochemical surface of sample “Ru/Ti-1” is larger than that of “Ru/Ti-3”. Additionally, we measured the activity of the samples in the oxidation and reduction of $K_{4}\left[\mathrm{Fe}{\left(\mathrm{CN}\right)}_{6}\right]$ (Fe${}^{+\mathrm{II}}$/Fe${}^{+\mathrm{III}}$), which is very consistent with the data from HER and OER. This investigation is performed to assess the principal possibility of the prepared samples to work in an electrochemical and electrocatalytic environment. The conducted experiments are not meant to be exhaustive, which would be beyond the scope of this work. The results of these measurements are compared to the activity of a Pt foil in the oxidation and reduction of Fe and are shown in Figure 22.

As expected, we cannot see any clear Fe redox features for either of the two sides of sample “Au” or for sample “Au/Pt”. This indicates that the uncoated stainless steel is inactive in the Fe redox reaction, and it is difficult to show the clear redox feature with such a low amount of Au or Pt particles. Therefore, it can be assumed that the low ability for transferring electrons to an electroactive species is the origin for the electrocatalytic inactivity for HER and OER. On the other hand, the redox feature is present in the case of the other samples and the Pt foil, with a peak separation of 60 mV, 88 mV and 61 mV for “Ru/Ti-1”, “Ru/Ti-3”, and the Pt foil, respectively.

**Figure 23:**
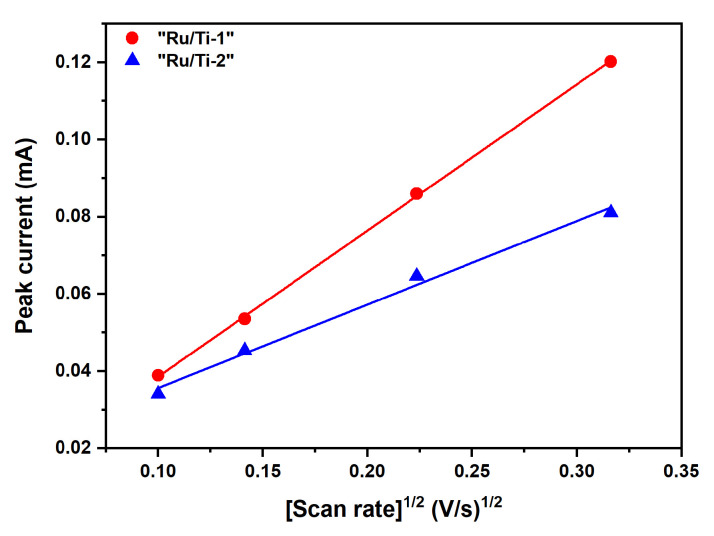
Peak current as a function of the square root of the scan rate for cyclyc voltammograms obtained in a solution containing 1 mM $K_{4}\left[\mathrm{Fe}{\left(\mathrm{CN}\right)}_{6}\right]$ in 0.1 M KOH. A higher slope indicates a higher electroactive surface area.

In Nernstian or reversible systems, the peak current is obtained by the Randles–Sevcik equation:

(1) $$I_{n}=kn^{2/3}AD^{1/2}Cv^{1/2},$$

with $k=2.686\cdot 10^{5}$ and $I_{n}$ being the peak current, *n* the number of electrons transferred in the redox event, *A* the area of the interface, *D* the diffusion coefficient of the transferred species, *C* the concentration, and *v* the scan rate. When considering this equation, we can easily appreciate that a higher slope implies a higher electrochemically acitve surface area. As can be seen in Figure 23, the slope for sample “Ru/Ti-1” is higher than that for sample “Ru/Ti-3”. The calculated active surface areas are 0.55 cm${}^{2}$ and 0.31 cm${}^{2}$ for samples “Ru/Ti-1” and “Ru/Ti-3”, respectively. Comparison of these results with the reported SEM, TEM, and XPS measurements leads to the assumption that the difference in the active surface area stems from different coverage of the Ru particles with ZrO${}_{2}$. The similar coverage of the substrate with Ru particles but a higher amount of (inactive) ZrO${}_{2}$ on the Ru particles would lead to a deterioration of the contact of the electrolyte with the active material, which again gives rise to the lower calculated active surface area.

## 5. Conclusions

We investigated four samples produced by a novel method for thermal spray deposition given in the patent [35]. This method is able to generate a variety of electrochemically active materials by depositing active metals (e.g., Au, Pt, Ru) on conductive substrates (e.g., stainless steel, Ti). However, the choice of substrate is limited to materials which can withstand the high temperatures generated during the thermal spray process. Additionally, the substrate as well as the operational mode of the method reported in the provisional patent have to be chosen carefully in order to obtain a substrate surface oxide layer of a size which does not influence the intended function of the electrocatalytic material. The same holds true for the diffusion of the catalyst material into the support material. Finally, the preparation of the catalyst material for the thermal spray process has to be performed carefully in order to avoid the deposition of residue material. In accordance with the provisional patent, various shapes and sizes of catalyst particles can be found. The preparation method enables the selection of particle coverage as exemplified by the different degrees of coating in the investigated samples. We tested the activity of all samples in the HER and OER and found that the two samples with Ru as the active phase exhibited a higher activity compared to the other two samples. The tested materials also showed a different activity towards the Fe${}^{+\mathrm{II}}$/Fe${}^{+\mathrm{III}}$ redox reaction which was used to make a first assessment of the electrochemical performance. As in the HER and OER, the two samples containing Ru showed an increased activity compared to the other samples. Differences in the calculated active surface area could be traced back to a varying ZrO${}_{2}$ coverage of the active Ru particles.

The described method provides a new and variable approach to the synthesis of supported electrocatalysts. Owing to the great variability of substrates and catalyst materials that are accessible with this technique, as well as the comparatively low temperature during deposition [32,35], this approach presents a useful addition to the established methods for electrocatalyst production. This method can be used for, e.g., the production of coated (bipolar) plates used in fuel cells or batteries. Due to the versatility of the described technique, it is possible to produce large plates (size in the range of square meters) covered with catalytic materials for various industrial applications.

## Figures and Tables

**Table 1:** Overview of the investigated samples. The sample description gives information about the support material (SS: stainless steel) and the catalyst material.

Sample Number	Sample Description	Support	Catalyst	Label in Text
25390	SS + Au	Stainless steel	Au	Au
25391	Ti + Au + Pt	Ti	Au and Pt	Au/Pt
25392	SS + Ru + Ti 39-81-1	Stainless steel and Ti	Ru	Ru/Ti-1
25393	SS + Ru + Ti 39-81-3	Stainless steel and Ti	Ru	Ru/Ti-3

## Supplementary Materials

The following are available online at https://www.mdpi.com/1996-1944/13/12/2746/s1, Figure S1: SEM images of sample “Au”, Figure S2: Additional EDX map of sample “Au”, Figure S3: STEM HAADF image of sample “Au”, Figure S4: SEM images of sample “Au/Pt”, Figure S5: Additional EDX map of sample “Au/Pt”, Figure S6: EDX linescan of the sample “Au/Pt”, Figure S7: Additional EDX map of sample “Ru/Ti-1”, Figure S8: STEM-EDX map of sample “Ru/Ti-1”, Figure S9: EDX spectra extracted from the different layers of the sample “Ru/Ti-1”, Figure S10: Additional EDX map of sample “Ru/Ti-3”, Figure S11: XP spectra of sample “Ru/Ti-3”, Figure S12: Cyclic voltammograms of all investigated samples.
