# Supplementary material for: Investigation of Electrocatalysts Produced by a Novel Thermal Spray Deposition Method
Source: Materials (Basel). 2020 Jun 17;13(12):2746. doi: 10.3390/ma13122746 (PMC7345183; doi:10.3390/ma13122746)
Supplement: Supplementary file 1 [file materials-13-02746-s001.pdf]

materials

Supplementary Information

Investigation of Electrocatalysts Produced by a Novel Thermal Spray Deposition Method

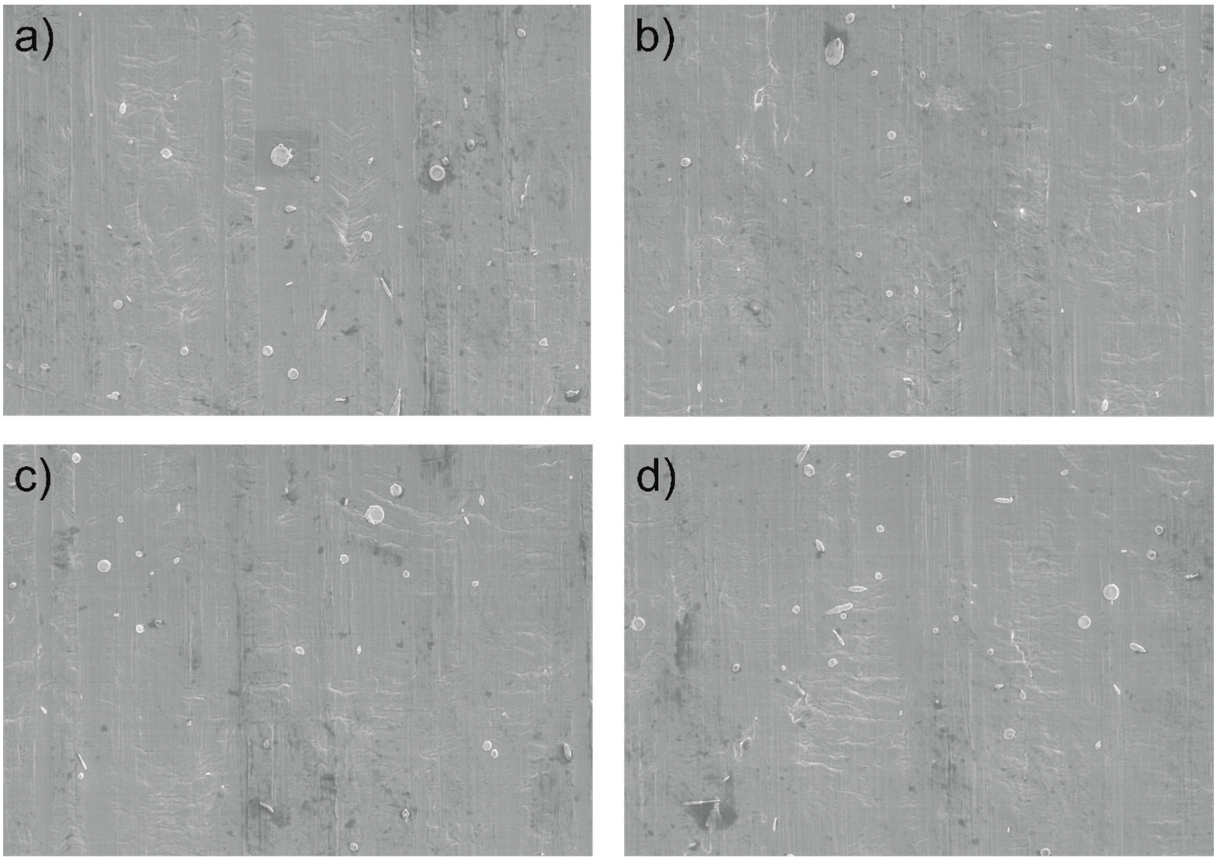

Figure S1. SEM images of sample "Au" that were used to analyse the particle size distribution.

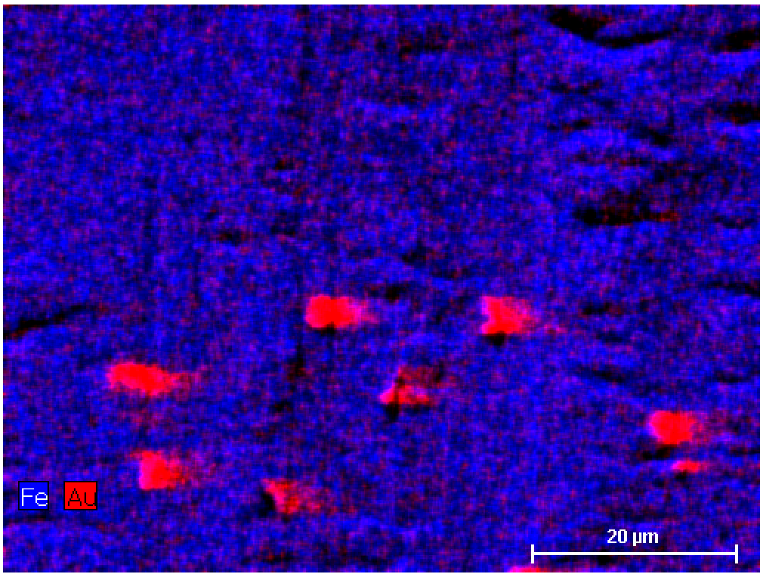

Figure S2. Additional EDX map of sample "Au".

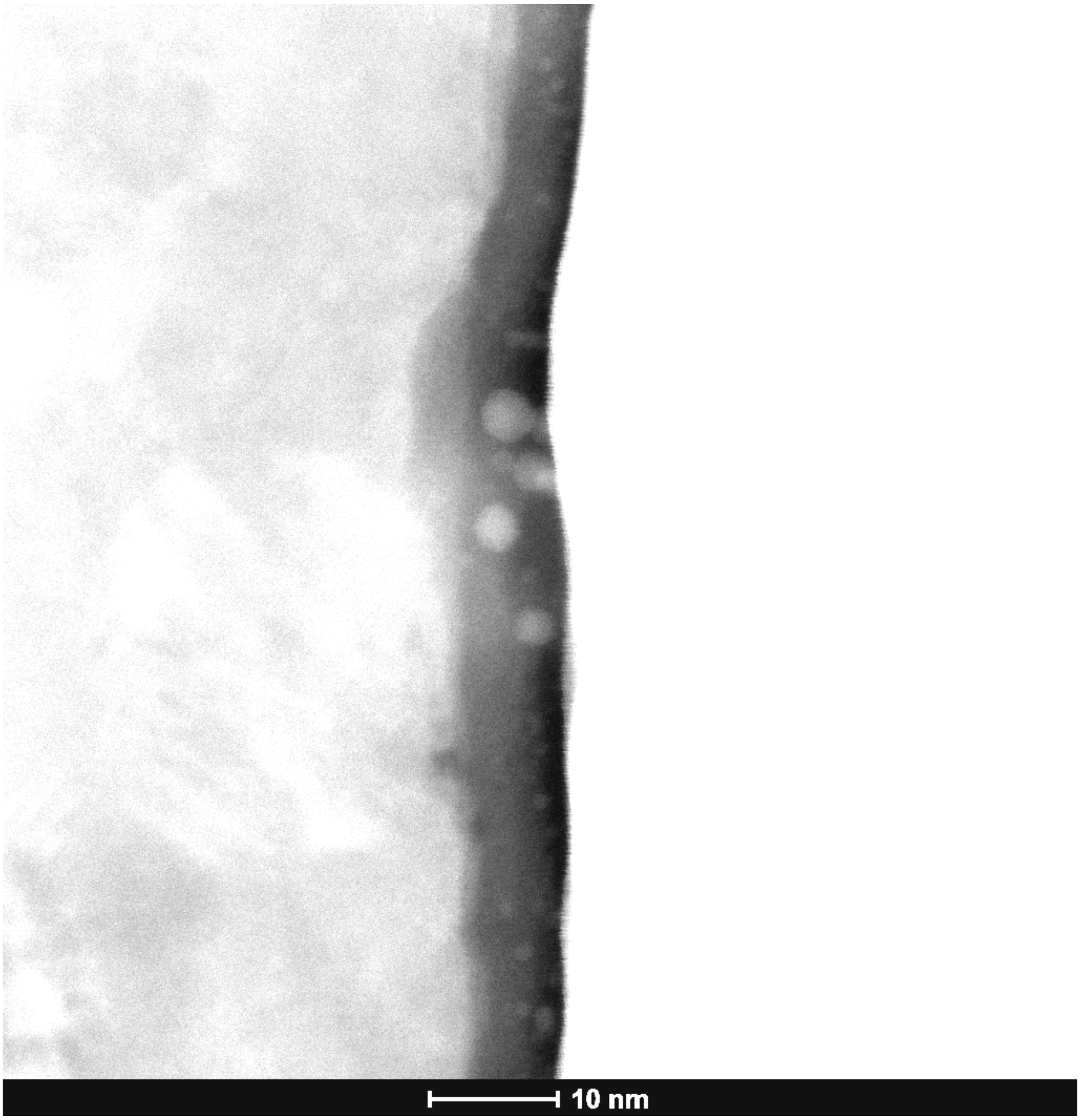

Figure S3. STEM HAADF image of sample "Au" indicating the presence of small Au nanoparticles in the interfacial layer between the steel substrate (left) and a larger Au catalyst particle (right).

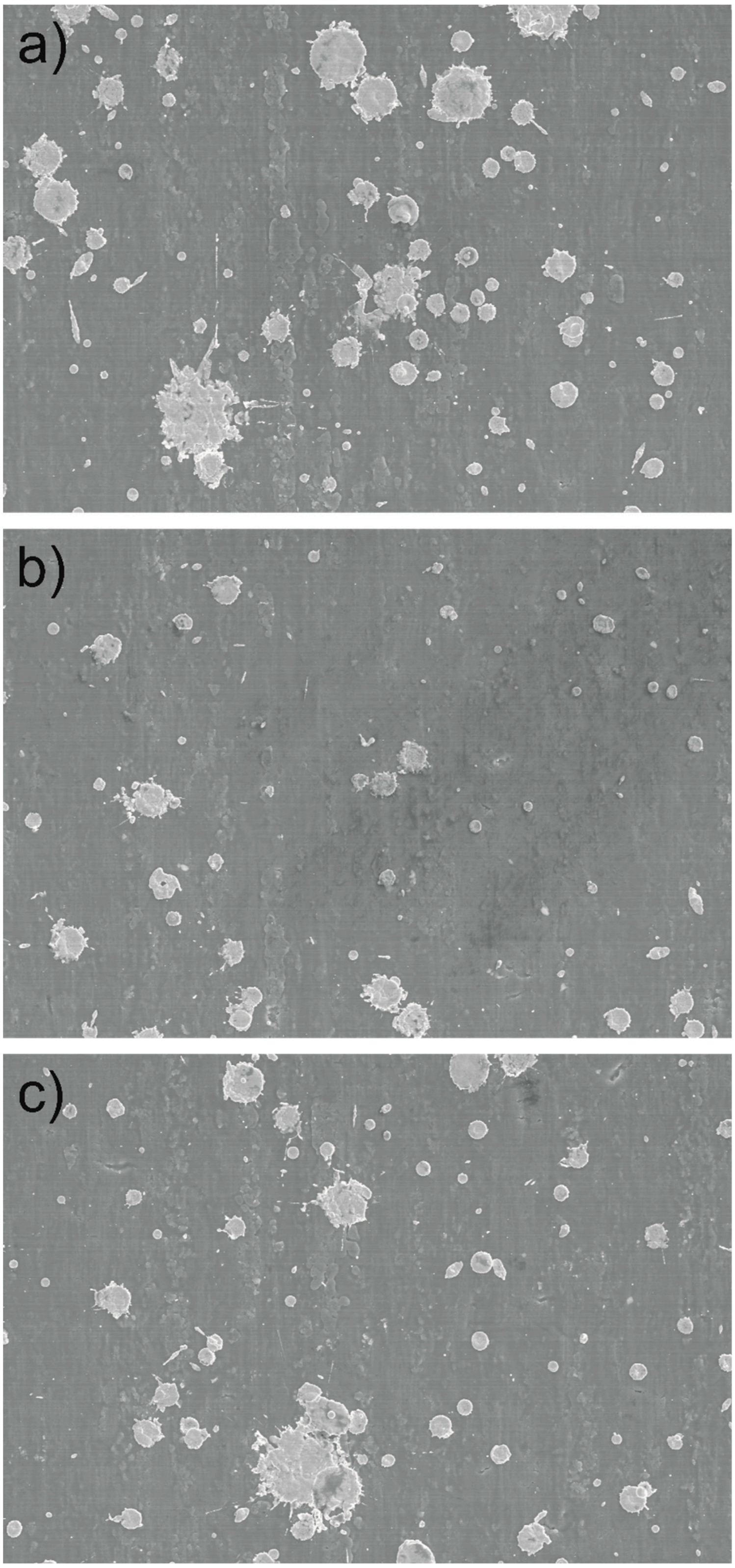

Figure S4. SEM images of sample "Au/Pt" that were used to analyse the particle size distribution.

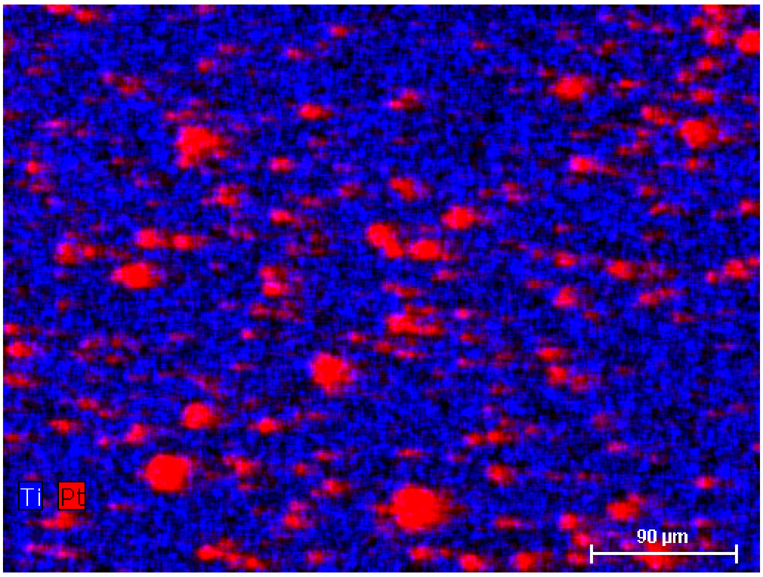

Figure S5. Additional EDX map of sample "Au/Pt".

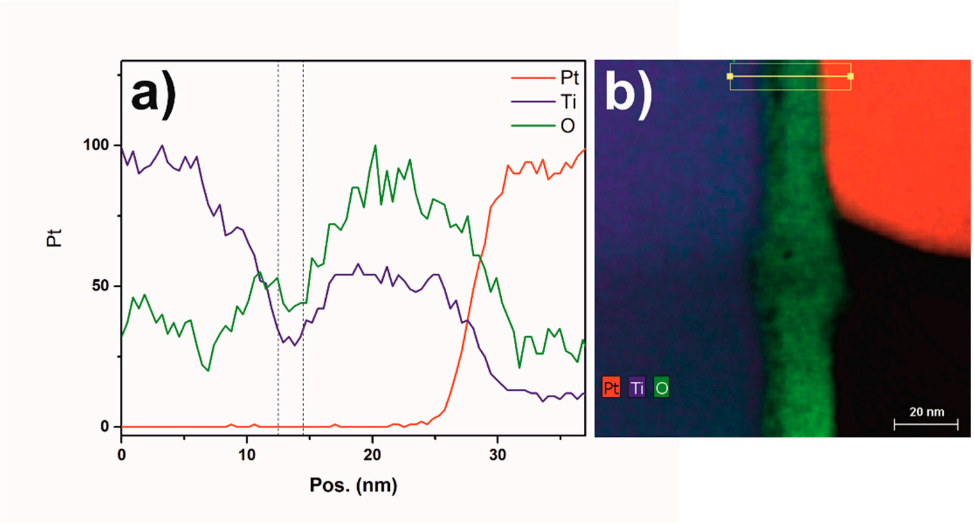

Figure S6. (**a**) EDX linescan of the sample "Au/Pt". In the linescan, between positions 12.5 nm and 14.5 nm, the region is marked in which a decrease in overall EDX intensity can be observed. (**b**) EDX mapping indicating the region from which the linescan was extracted.

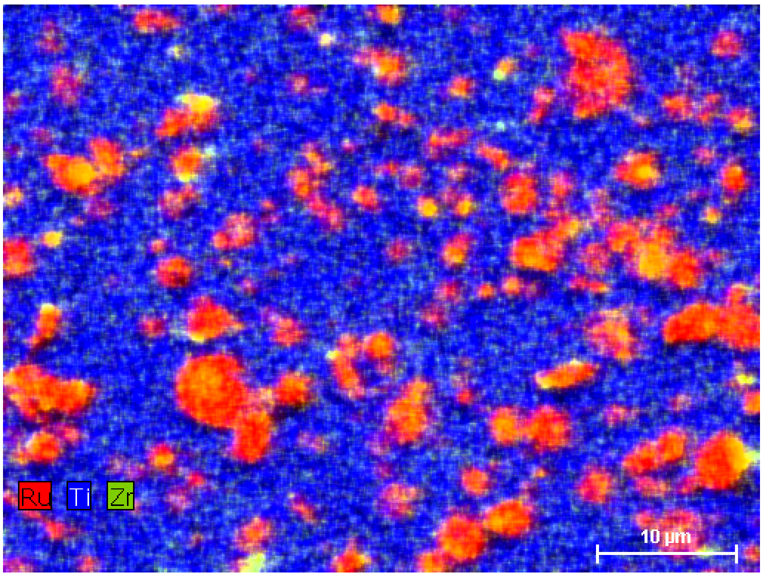

Figure S7. Additional EDX map of sample “Ru/Ti-1”.

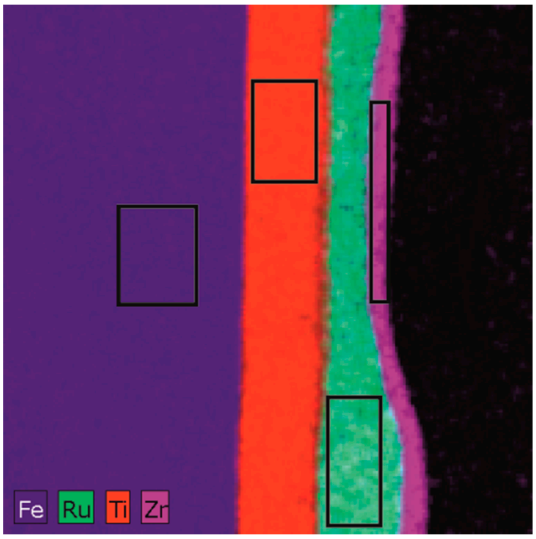

Figure S8. STEM-EDX map of sample “Ru/Ti-1”. The regions from which the EDX spectra shown in Figure S9 were extracted are marked in the map.

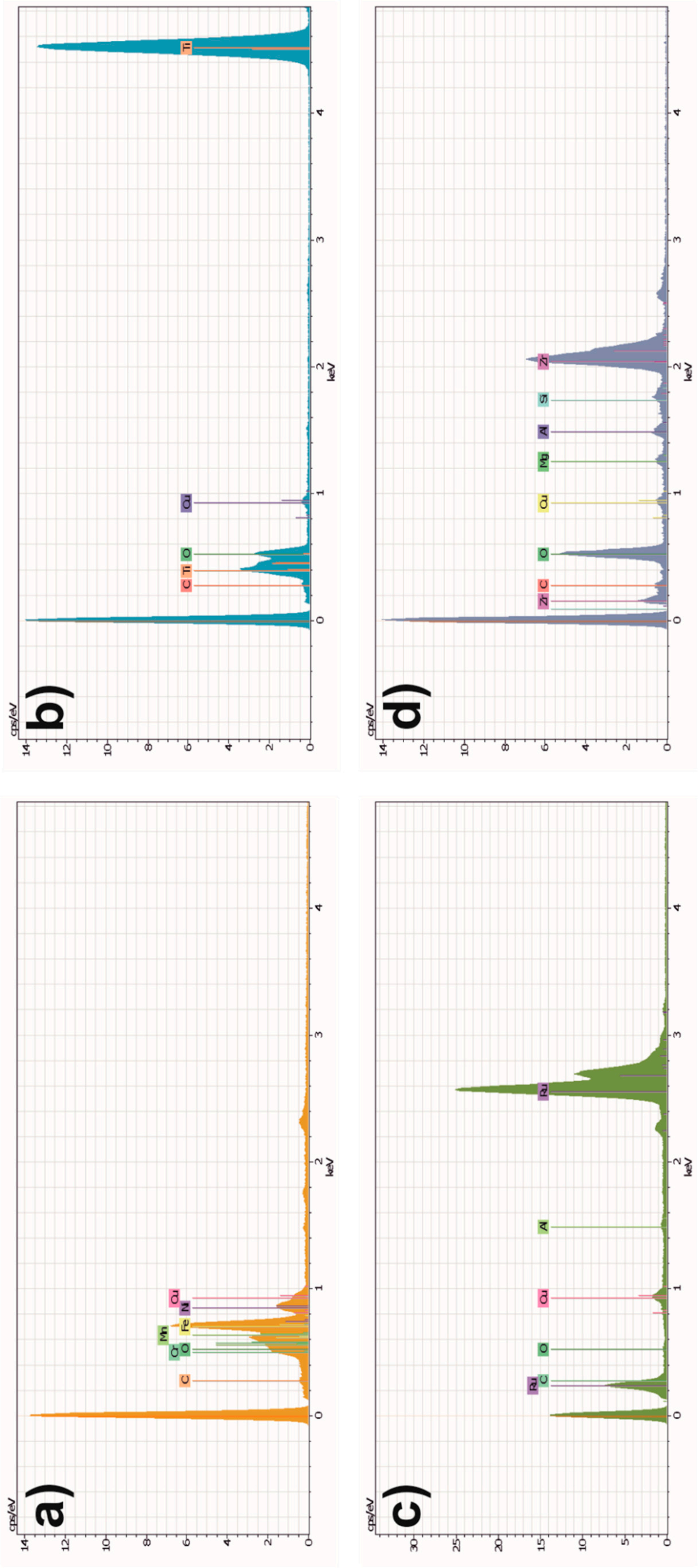

Figure S9. EDX spectra extracted from the different layers of the sample "Ru/Ti-1" as marked in Figure S8. (**a**) EDX spectrum from the steel layer, (**b**) EDX spectrum extracted from the Ti-layer, (**c**) EDX spectrum from the Ru-layer and (**d**) EDX spectrum extracted from the Zr-layer.

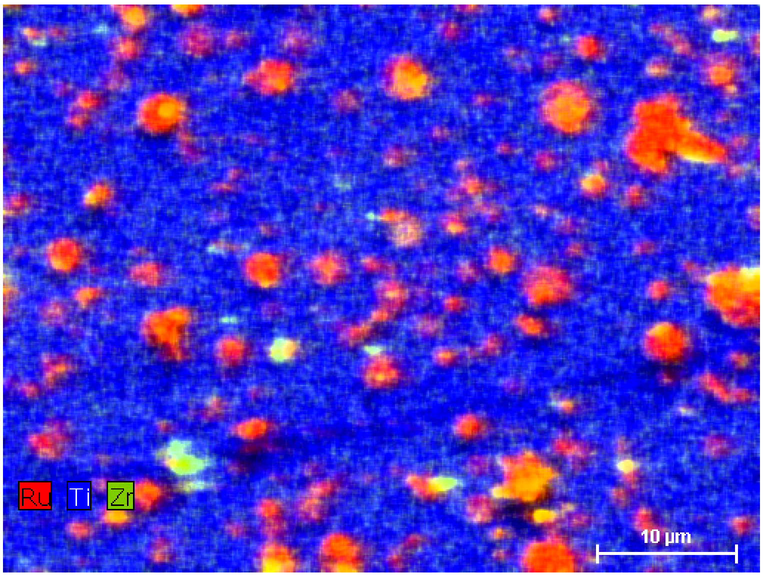

Figure S10. Additional EDX map of sample "Ru/Ti-3".

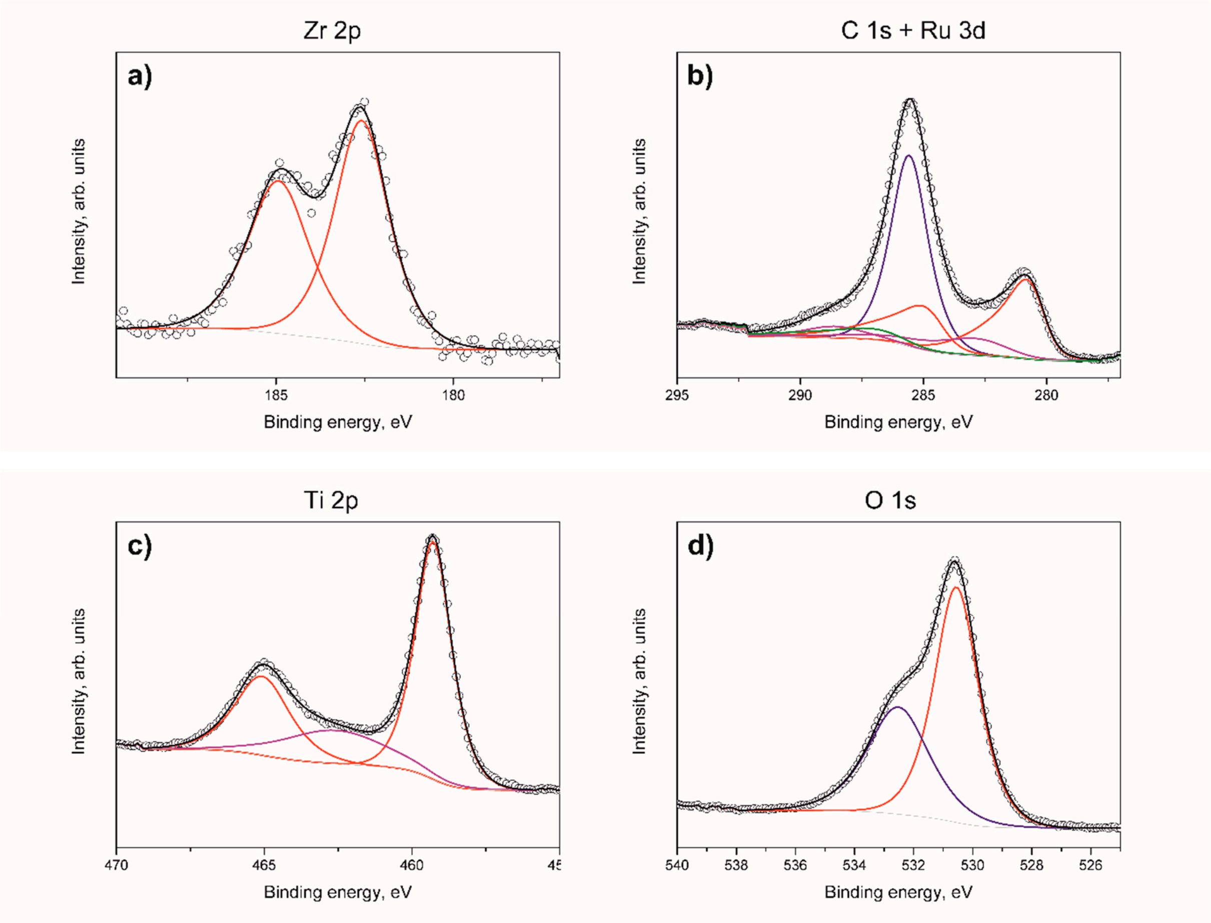

Figure S11. XP spectra of sample "Ru/Ti-3". The peaks of (**a**) Zr 2p, (**b**) C 1s and Ru 3d, (**c**) Ti 2p and (**d**) O 1s and corresponding fits are shown.

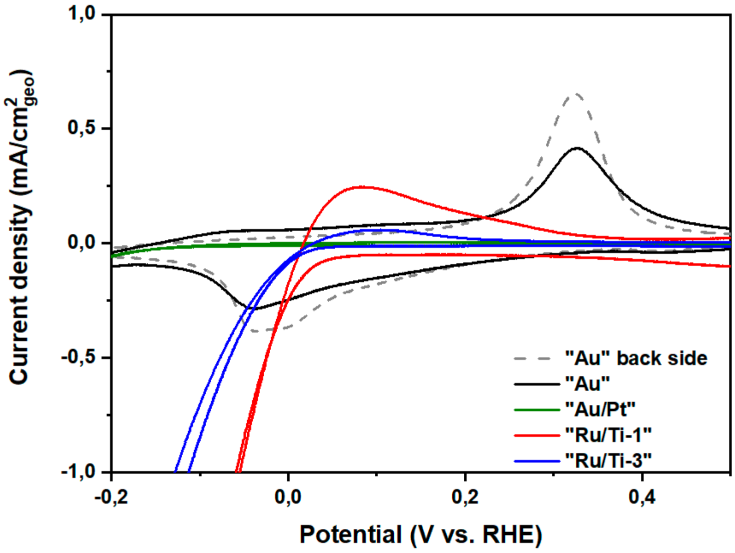

Figure S12. Cyclic voltammograms of all investigated samples obtained at a scan rate of 20 mV/s in Ar saturated 0.1 M KOH. This graph focusses on the redox peaks in the region from -0.1 V to 0.4 V which can be attributed to the oxidation/reduction of $Fe^{+II} \leftrightarrow Fe^{+III}$. It has to be noted that the back side of sample "Au" is pure stainless steel. The peaks are well visible for the sample "Au" which consists of Au catalyst particles on a stainless steel substrate with a coverage of only 0.57%. Therefore, a strong contribution of the substrate is expected and visible.

© 2020 by the author. Licensee MDPI, Basel, Switzerland. This article is an open access article distributed under the terms and conditions of the Creative Commons Attribution (CC BY) license (http://creativecommons.org/licenses/by/4.0/).
